# Diverse Applications of Marine Macroalgae

**DOI:** 10.3390/md18010017

**Published:** 2019-12-24

**Authors:** Adriana Leandro, Leonel Pereira, Ana M. M. Gonçalves

**Affiliations:** 1 MARE (Marine and Environmental Sciences Centre), Department of Life Sciences, Faculty of Sciences and Technology, University of Coimbra, 3004-517 Coimbra, Portugal; 2 Department of Biology and CESAM, University of Aveiro, 3810-193 Aveiro, Portugal

**Keywords:** macroalgae/seaweed, natural resources, health, food, feed, agriculture

## Abstract

The aim of this paper is to review the multiplicity of the current uses of marine macroalgae. Seaweeds are already used in many products and for different purposes, from food products to medicine. They are a natural resource that can provide a number of compounds with beneficial bioactivities like antioxidant, anti-inflammatory, anti-aging effects, among others. Despite studies directed in prospecting for their properties and the commodities already marketed, they could, surely, be even more researched and sustainably explored.

## 1. Marine Macroalgae Diversity and Ecology

The marine environment is home for many diverse organisms such as algae, molluscs, sponges, corals, tunicates. Currently, oceans are already considered the “lungs of the Earth” due to Cyanobacteria and other algae that live in seawater. In fact, these beings provide up to 80% of the atmospheric oxygen, which we rely on to breathe. Cyanobacteria are blue colored, aquatic, photosynthetic, and because they are bacteria, they usually are unicellular, but they often grow in colonies large enough to be seen. Cyanobacteria are prokaryotic organisms while algae are eukaryotic organisms. Algae are almost ubiquitous, between microscopic and macroscopic species, they can be found in every wet environment in land, in fresh water or in oceans [1]. 

In this review, the focus is the marine macroalgae or seaweeds, which are multicellular, macroscopic, eukaryotic, and autotrophic organisms. They are taxonomically organized in three large and distinct groups, based on the color of the thallus: Chlorophyta (green algae), Rhodophyta (red algae), and Ochrophyta – Phaeophyceae (brown algae). All of them accumulate starch in the interior of their cells as energy store, and other different polysaccharides of large molecular chain. The green algae produce ulvan and contain carotene and xanthophylls and chlorophylls *a* and *b* (what sustains the idea that they are the ancestors of the plants) as pigments. The red algae (most common in hot seas) have chlorophylls *a* and *d* and carotenoids and their staining is due to the presence of phycoerythrin (pigment) in their cells. In the brown algae are found the pigments fucoxanthin, chlorophylls *a* and *c* and carotenoids and, as reserve substances, oils, and polysaccharides (such as laminarin) [2,3].

Like plants in terrestrial land, seaweeds have similar ecological roles but in aquatic territory. Some macroalgae species may serve as bioindicators of the quality of water and some can do bioremediation by bioabsorption and bioaccumulation [4,5,6].

As other vegetables, seaweeds are primary producers, the base of the marine food chain, sustaining several benthic animal communities [7]. They also compete for light, nutrients, and space, in addition to the need of carbon dioxide and water to develop. Inclusively algae and plants produce the same storage compounds and use similar defence strategies against predators and parasites [2]. They have also developed effective mechanisms to survive many biotic threats, like bacteria, virus, or fungal infections. Because they are sessile organisms, seaweeds have evolved to live in variable, extreme, and hostile abiotic environmental and stress conditions, like temperature changes, salinity, environmental pollutants, or UV radiation exposure. That caused these beings to be able to produce a wide range of compounds called ‘secondary metabolites’, like pigments, vitamins, phenolic compounds, sterols, and other bioactive agents. Besides these, they also produce amino acids and proteins, saturated/unsaturated fatty acids and all kinds of polysaccharides which are directly implicated in the development, growth, or reproduction conditions to perform physiological functions. So, based on the production of these molecules, in addition to its ecological importance, marine macroalgae also have great importance at commercial level. That’s why, a few years ago, the interest in the cultivation and exploitation of macroalgae in the most varied forms increased. Seaweeds are already used in many countries for very different purposes, like industrial phycocolloids extraction or extraction of compounds with antiviral, antibacterial, or antitumor activity [8]. They can also be, directly or indirectly, used for human and animal nutrition (livestock) or farming (biofertilizers) [9]. 

**Figure 1:**
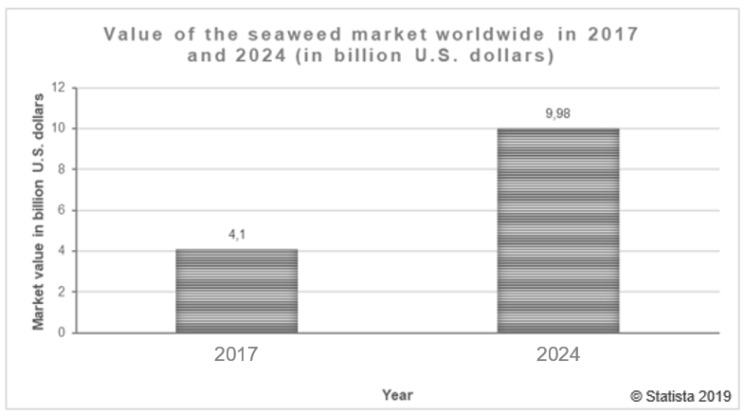
Value of the seaweed market worldwide in 2017 and 2024 (in billion U.S. dollars) [10].

Although there is still much to investigate and find out about these living beings, it is known that several of the substances they synthesize have great potential to be used in areas such as pharmaceutical, cosmetics and the food industry. As their interest, cultivation and applications increase, their value in the market rises too. It is estimated that in 2024 this value will exceed twice the achieved in 2017 (see Figure 1) [10].

## 2. Marine Macroalgae Applications 

### 2.1. Human Food

Asian countries, especially China and Japan, are known for being large seaweed consumers for many centuries. The first records show that the harvesting of macroalgae, such as *Laminaria* spp., *Undaria pinnatifida*, *Sargassum fusiforme* (formerly *Hizikia fusiforme*) (commonly known as kombu, wakame, and hiziki, respectively), for human consumption was already carried out by people in China, at least from 500 B.C. [7]. While in Europe it occurred a thousand years later [9]. 

More than 600 species of edible seaweeds are categorized. Now it is recognized that edibles seaweeds have great nutritional content as they are a low caloric food, but rich in vitamins, minerals, and dietary fibers [7,9]. Their nutritive value may vary depending on the geographic location, season of the year, growth stage, part of the seaweed harvested, etc [11]. Thus, to assure the nutritional value of seaweeds, they need to be evaluated before being used as supplements.

Seaweeds draw from the sea an incomparable wealth of mineral elements, macro elements, and trace elements. They are known as an excellent source of vitamins and minerals, especially potassium and iodine (i.e., *Palmaria palmata*, *Fucus vesiculosus*, *Laminaria* sp.), and potentially good sources of proteins (i.e., red algae such as *Pyropia tenera* (formerly *Porphyra tenera*), *Grateloupia filicina*), long-chain polysaccharides (i.e., *P. tenera*) and soluble and insoluble dietary fibers (i.e., *G. filicina*, *Chondrus crispus*, *Ulva lactuca*) [12]. It was found that the ashes of edible seaweeds contained higher amounts of macrominerals (8.083–17.875 mg/100 g; Na, K, Ca, Mg) and trace elements (5.1–15.2 mg/100 g; Fe, Zn, Mn, Cu), than those reported for edible land plants. So edible brown and red seaweeds could be used as a food supplement to reach the recommended daily intake of some essential minerals and trace elements [13]. For example, the consumption of 10 g of the green alga *Ulva lactuca* provides 70% of the body’s daily magnesium requirements and over half of its iron requirements [12,13].

Macroalgae can be used like other vegetables, being equally or even more versatile than them. Sea vegetables, as they are known, could be commercialized and/or eaten in many forms, such as fresh, dried, in flakes, flour or powder, or incorporated in other food products (added-value products) (see Table 1). 

The entire organism can be eaten freshly harvested or after dried and then re-hydrated and cooked [7,16]. They are already commercialized under multiple brands, and labeled with “fat-free”, “gluten-free”, “mineral rich”, “low carbohydrates”, and “low calories” [14,15,18,21]. There are natural and healthier substitutes of pasta or bacon (i.e., *Himanthalia elongata*, as spaghetti, and *Palmaria palmata*, as sea bacon, both from Seamore food company), the well-known nori sheets (genus *Porphyra*/*Pyropia*) to prepare sushi rolls or crispy thin snacks, and many other recipes such as wraps with *Undaria pinnatifida* (wakame) and *H. elongata*, or the laverbread, a paste prepared with boiled nori (also recognized as laver) [15,18] and in desserts like in innovative Spanish nougats with crushed nori algae [21]). Above all this nutritional value, macroalgae are donors of a number of great ‘side-effects’ acting as nutraceuticals. A study with Japanese children revealed that seaweeds intake in the diet was significantly negatively related to systolic blood pressure in girls and with diastolic blood pressure in boys. This study suggests that seaweeds have beneficial effects on blood pressure [22].

As we can see, macroalgae can be incorporated in food products after processed into flakes, flour, powder or even in more specific extracts. Their pigments, like carotenoids, are, in fact, one of the products of interest for the food industry [23]. Traditionally, carotenoids have been used in that industry due to their properties as natural color enhancers. However, those that are synthetically obtained are now suspected of being promoters of carcinogenesis and liver and renal toxicity. So, there is a strong market demand to replace them with natural pigments. Seaweeds are a great source of many pigments, especially β-carotene which besides its anticancer activity, has been reported that it is absorbed 10 times more easily by the body than the synthetic one [24].

Pigments are important in this industrial range, but there are some constitutes of algae that are even more, their hydrocolloids, such as carrageenan, alginic acids, and agar. These are the main constituents of red and brown algal cell walls and are widely used in several food industries (see Table 2). 

Carrageenan is a natural phycocolloid and is one of the main additives used by the food industry, in many dairy products (e.g. yoghurts, flavoured milkshakes, flans, jellies, ice creams, and beers) and meat products (e.g. hams), as thickening, emulsifier or stabilizing agent [3,25,26]. Extracted from several families of the order Gigartinales. These polygalactans are sulfated and have a linear structure formed by galactose residues with alternating α (1–3) and β (1–4) bonds. Regulatory authorities (FDA) have established a minimum value for the molecular weight of the carrageenan to be used in food preparations. The commercial carrageenans usually range from 400 to 600 kDa, having the minimum of 100 kDa. This minimum value was established by the response to reports of highly degraded carrageenan-induced ulceration of the colon. There are three main varieties of carrageenan, differing in their sulfation degree. Kappa (κ)-carrageenan has one sulfate group per disaccharide, Iota (ι)-carrageenan has two sulfates and Lambda (λ)-carrageenan has three sulfates per disaccharide. The type of carrageenan selected is dependent on the desired finished product characteristics. Iota and kappa carrageenans are gelling carrageenans, while lambda is a thickening/viscosifier carrageenan [28].

Agar is other phycocolloid, composed of a variable combination of agarose and agaropectin, depending of the species and seasonal factors. Agarose, which is the primary component of agar, is a linear polymer of agarobiose, a disaccharide composed of D-galactose and 3,6-anhydro-L-galactopyranose. Agaropectin, which occurs usually in minor amounts, is a heterogeneous mixture of β-1,3-linked D-galactose which contains substituted sulfate and pyruvate moieties. Like carrageenan, agar has a similar application, so it also has gelling properties, but while carrageenan gels by both ionic and hydrogen bonds, agar gels only by hydrogen bonds. Extracted from several species of red algae, mainly the *Gelidium* sp., *Gracilaria* sp. and *Pterocladiella* sp., it is frequently used as thickener in food products and a vegetarian substitute for gelatine [7,9]. Currently, agar is also being used to develop a new biomaterial for packaging. Made from agar and other natural raw materials, these new wrappers are sustainable, biodegradable, and constitute an alternative to plastics [29]. 

Alginate is also a gelling agent found and extracted from brown seaweed (eg. *Ascophyllum* sp., *Laminaria* sp., *Lessonia* sp., *Macrocystis* sp.). In fact, it is the most abundant marine biopolymer and, next to cellulose, the most abundant biopolymer in the world. Alginate is a linear acidic polysaccharide that can be a homopolymer or a heteropolymer of β-d-mannuronate and/or α-l-guluronate [27]. It is used as a stabilizer in many food products like ice cream, yogurt, cream, and cheese. It is also used in the food industry as a thickener, emulsifier for sauces, dressings, and jam, and it needs no heat to gel. It is most commonly used with calcium lactate or calcium chloride in the spherification process, a technique performed in molecular cooking.

### 2.2. Livestock and Agriculture

The consumption of macroalgae is not only for humankind, but also for other animal species. European usage of seaweeds in animal husbandry has come since the time of the Romans. Countries such as Iceland, France, and Norway usually use them in domestic animal nutrition [30]. In fact, the first seaweed meal for animal feed was produced in Norway. It was made from brown seaweeds that were collected, dried, and milled [7]. 

Besides its direct uses as feed, macroalgae are already introduced in other type of feed as a nutritive additive, and as a nutraceutical compound. Currently the feeding of the animals is supplemented by algae to fill the deficiency in mineral pastures in the U.S.A., Australia and New Zealand. Seaweed meal, used principally as a vitamin and mineral supplement, is produced mainly from the kelps *Ascophyllum nodosum*, *Fucus* spp., *Laminaria* spp., *Macrocystis* spp. [30].

Extracts like macroalgae-derived sulfated polysaccharides are added to animal feed. It was proven that these meals can improve animal intestinal integrity and efficient immune response [31].

On the other hand, feeding seaweeds and macroalgal products has been shown to reduce enteric methane emission from rumen fermentation, [32] which makes this type of feeding a promising candidate as a biotic methane mitigation strategy in the largest milk or beef producing [33].

Moreover, seaweed and seaweed-derived products have been widely used in agriculture to improve crop production systems due to the existence of a number of plant growth-stimulating compounds [34,35,36]. Inclusively, since ancient times, they were traditionally used to fertilize the fields, they have long been used to augment plant productivity and food production in various regions of the world [30]. 

Seaweeds and their compounds can promote early seed germination, root and plant growth, confer tolerance to freezing, resistance to biotic stresses, and increase the plant nutrient absorption capacity [37,38]. For example, auxins, a plant hormone responsible for the vegetative growth, and auxin-like compounds were detected in some seaweeds [39].

However, the biostimulator potential of many of these compounds has not been fully exploited due to the lack of scientific data on growth factors present in seaweeds and their mode of action in affecting plant growth [36]. The effects are complex and dependent on the crop, the local environmental conditions and on the interactions of the algae species with the soil community [30]. Seaweeds’ extracts, like laminarin, have been shown to stimulate natural defence responses in plants and are involved in the induction of genes encoding various pathogenesis-related proteins with antimicrobial properties. Also, it has been demonstrated that alginate oligomers show growth-promoting effects on certain higher plant species [35].

Studies suggest that adding strongly polar degraded fucoidan, alginate, etc., to soils improves crumb structure and aeration, thus stimulating microorganisms and root systems which improves plant growth [40]. 

The current commercial extracts are manufactured mainly from the brown seaweeds *Ascophyllum nodosum*, *Laminaria* spp., *Saccorhiza* spp., *Ecklonia maxima*, *Fucus* spp., *Sargassum* spp., and *Durvillaea* spp., although other species such as *Ulva intestinalis*, *Ulva lactuca*, *Codium* sp. (Chlorophyta), *Gelidium* sp., and *Chondrus crispus* (Rhodophyta) are also used [30].

### 2.3. Cosmetics

The definition of cosmetic product, according to the European Commission, is: “Any substance or mixture intended to be placed in contact with the external parts of the human body (epidermis, hair system, nails, lips, and external genital organs) or with the teeth and the mucous membranes of the oral cavity with a view exclusively or mainly to cleaning them, perfuming them, changing their appearance, protecting them, keeping them in good condition, or correcting body odours” [41].

More recently, there is another category—the ‘cosmeceuticals’—which is attracting the industry’s attention and is of interest to the most attentive consumers. Despite still being without legal meaning nowadays, the industry continues to use this designation referring to a product that lies between the benefits of cosmetics and pharmaceuticals [42,43].

There is a growing demand for more natural cosmetics, those made with natural/organic ingredients, due to the benefits they offer, and the absence of many harmful chemicals which are present in conventional cosmetics products. Consequently, the cosmetic industry is rapidly expanding to meet these increased demands. Some of the key active-based natural ingredients used in cosmetics are extracted from marine organisms, like seaweeds (see Table 3). 

Marine macroalgae are one of the most abundant sources of vitamins, minerals, amino acids, antioxidants, and essential fatty acids. Seaweeds are unique in containing bioavailable ingredients, meaning that its active, nutrient-rich compounds are more readily absorbed by the skin and the body. Because of its bioavailable nature, seaweeds provide a multitude of benefits including reducing the appearance of redness and blemishes, brightening, hydrating, re-mineralizing, reducing the appearance of sun damage, and firming skin [44,45,46].

Algae can be incorporated into these products as algal extracts of selected elements or, alternatively, pieces of dried seaweeds can be crushed and ground and incorporated into skin care products such as exfoliating lotions, face masks, face washes and soaps. Cosmetic products, such as creams and lotions, sometimes show on their labels that the contents include “marine extract”, “extract of alga”, “seaweed extract”, or similar [7,44]. Usually this means that one of the hydrocolloids extracted from seaweeds was added to the product. Alginate or carrageenan are water-binding agents, which means they help hold water onto the skin and hair, increasing the moisture balance [47]. Both can be found in multiple products like lotions, creams, shampoos, conditioners, and toothpastes [48]. 

Seaweeds can be used in two ways in cosmetics: they can either be a vehicle, serving as a stabilizing, emulsifying, or other type of agent necessary for product preparation; or as the active therapeutic ingredient in the product, for example in anti-aging skin treatments or after-sun skin care products [13,48,49]. 

Algae are rich in saturated and unsaturated fatty acids that are bioactive compounds. For example, palmitic acid and other fatty acids, that are present in large quantities in marine seaweeds, are used in cosmetics as emulsifiers, and its derivated ascorbyl palmitate is an antioxidant that is effective for anti-aging and anti-wrinkle effects [48,50]. 

Purified phlorotannins extracted from brown seaweeds are included in cosmetics, since these molecules have the role of preventing and slowing down the skin aging process, which is mainly associated with free radical damage and with the reduction of hyaluronic acid concentration [51]. 

Wang et al. [52] compared the moisture-absorption and retention properties of polysaccharides extracts from five different seaweed species [52]. Marine algae are reported to produce different polysaccharides, including alginates, ulvans, laminarans, and fucoidans [53]. These molecules usually contain large proportions of L-fucose and sulfate, together with minor amounts of other sugars such as xylose, galactose, mannose, and glucuronic acid [45]. In their study, Wang et al. [52] reported that the polysaccharides extracted from brown seaweed (more precisely the fucoidan obtained from the *Saccharina japonica*) exhibited the best moisture-absorption and retention capacity, while the green ones were the worst. This ability of polysaccharides is influenced by its sulfated content, molecular weight (length of chain), and by the type of algae that they are extracted from [52]. An example of it is a cosmetic, CODIAVELANE^®^, composed of propylene glycol, water, and *Codium tomentosum* extract. It is proven that it normalizes and balances skin’s moisture content by adding oligo-elements and increasing surface hydration [49].

A group of small water-soluble compounds, mycosporine-like amino acids (MAA), found in marine algae, is biologically relevant because of its photo-protective potential. In addition, its antioxidant and skin protective strategies raise the interest for possible pharmaceutical and cosmetic applications [54,55]. An extract of *Asparagopsis armata* (ASPAR’AGE™) containing this MAA molecules is already incorporated in some lotions with anti-aging properties [56]. 

Besides the numerous existing and marketed cosmetics and cosmeceuticals, there are many other seaweed extracts that are under investigation. Kamei et al. [57] discovered a compound from *Sargassum macrocarpum*, Sargafuran, that was bactericidal and completely killed Propionibacterium acnes by lysing bacterial cells [57]. The results suggest that this substance could be applied in new skin care cosmetics to prevent or improve acne.

### 2.4. Pharmaceutics 

The overuse of antibiotics can lead to the development of resistant pathogenic bacteria. New antibiotics that are effective against new and resistant bacterial strains are needed. As previously mentioned, seaweeds have evolved to survive many environmental stresses and threats. Besides the predators/herbivores, they have to continuously face high concentrations of infectious and surface-fouling bacteria that are indigenous to ocean waters [8]. So, the macroalgae have evolved and developed certain mechanisms of defence like the production of bioactive compounds. Substances such as phlorotannins, polysaccharides, and peptides allow seaweeds to avoid bacterial invasion [8], and some have been investigated about other potential pharmacological effects (antiviral, antitumoral, immunogenic effects). One example is the peptide kahalalide F and its isomer, iso-kahalalide F, extracted from a green macroalga, *Bryopsis pennata*, which present cytotoxic effects and were used in anticancer clinical trials. Despite its great potential, this molecule is under modification tests to improve its water solubility, stability, and effectiveness [70]. 

Sometimes the extract used can be obtained from a mix of various algae species, and even of different seaweed groups. For example, there is a patent of green and/or brown seaweed extract for the treatment of type 2 diabetes and its complications. This has brown seaweeds such as *Fucus vesiculosus* or *Ascophylum nodosum* and green algae, selected from the group consisting of *Cladophora* sp., *Monostroma* sp., *Ulva compressa* (as *Entoromorpha compressa*), *Codium* sp., among others [71].

According to another study, methanolic extracts of some brown, red and green algae are effective at inhibiting the growth of pathogenic Gram-positive (*Staphylococcus aureus*, *Micrococcus luteus*, *Enterococcus faecalis*) and Gram-negative bacteria (*Enterobacter aerogenes*, *Escherichia coli*) [72]. The species were *Corallina officinalis* (Rhodophyta), *Cystoseira barbata*, *Dictyota dichotoma*, *Halopteris filicina*, *Cladostephus spongiosus* (Ochrophyta, Phaeophyceae), and *Ulva rigida* (Chlorophyta). 

The seaweed-derived substances that received most attention from pharmaceutical companies are the sulfated polysaccharides (negatively charged sugar polymers due to the presence of sulfate groups). Sulfated polysaccharides are extracted from red algae (carrageenans and agarans), brown algae (e.g. fucoidans) and green algae (e.g. ulvans). Their value lies on their bioactivities, namely their antibacterial, antiviral activity, antitumoral, and immunomodulatory potential [8,70,72,73]. 

On the other hand, other polysaccharides, like alginate, are also used in pharmaceutical formulations as excipients. Alginate polymers have a wide potential in drug formulation due to their lack of toxicity and they can be tailor-made to suit the demands of applicants in both the pharmaceutical and biomedical areas. This brown seaweed—derived group of polymers owns a few characteristics that makes it useful as a formulation aid, both as a conventional excipient and more specifically as a tool in polymeric-controlled drug delivery [8,74], and it is commonly used as bio-adhesive in pharmaceutical applications [75]. Other application of alginate is in wound healing dressing due to the excellent swelling properties and biocompatibility [76]. In fact, not only the alginate, but seaweed extracts—like the *Laminaria* spp.—are being studied and used for the development of biodegradable wound care products, since they contain healing accelerator substances: alpha keto isovalerate, alpha keto glutarate, and alpha keto oxaloacate [77]. 

It has been demonstrated that alginate has therapeutic effects in mammalian systems such as anticoagulants and antitumor activities. Also containing alginate, there is some gastrointestinal formulations and protectors (i.e., Gaviscon), that neutralize the acids, prevents the contact of stomach contents with the oesophagus (reflux), and relieve symptoms of heartburn and indigestion [78]. 

Agar, which was initially used as a laxative agent in the preparation of medicines, in western countries [26], is now used as an ingredient in tablets and capsules, as well as in different types of emulsions. Like alginate, the main role of agar in the pharmaceutical industry is as an excipient.

The three main types (ι, κ, λ) of carrageenan form thermo-reversible gels in aqueous solutions and in the presence of cations. Therefore, they are used in pharmaceutical formulations for stabilization of disperse systems and viscosity modification [75]. In addition to its hydrating properties, it has also been found in some studies to block the growth of viruses like human papillomavirus, making it potentially even more protective in sexual lubricants (in which it is already included). Studies in vitro demonstrated that carrageenan, even when diluted a million-fold, presents activity against a range of common sexually transmitted HPV types that can cause cervical cancer and genital warts [47]. So, due to these properties, carrageenans might have a great interest in the composition of sexual lubricant. Also the polymer galactofuran (extracted from *Undaria pinnatifida*) was proven as an effective Herpes virus inhibitor [79]. 

Among polysaccharides, fucoidans were particularly studied as they showed interesting biological activities (anti-thrombotic, anti-coagulant, anticancer, anti-proliferative, and anti-inflammatory) [66,80,81,82]. 

Other group of small molecules (previously indicated in this article), the MAAs, have skin protective and wound healing effects. Like the porphyra-334 was able to suppress ROS (reactive oxygen species) production in human skin fibroblast cells [83]. Pigments isolated from seaweeds also have bioactivities. Like the fucoxanthin, obtained from *Saccharina japonica* (as *Laminaria japonica*), that has been reported to suppress tyrosinase activity in UVB-irradiated guinea pig and melanogenesis in UVB-irradiated mice. Oral treatment of fucoxanthin significantly suppressed skin mRNA expression related to melanogenesis, suggesting that fucoxanthin negatively regulated melanogenesis factor at transcriptional level [45]. 

Seaweed phlorotannin extracts from *Ascophylum nodosum* are reported to have potential in the treatment of diabetes [84] while those from *Ecklonia cava* are now marketed for potential health benefits due to their antioxidant activities [85]. These phlorotannins are phenols structurally different from those obtained from plants, since these are oligomers and polymers of phloroglucinol (1,3,5-tri-hhydroxybenzene) and the terrestrial ones are based on gallic acids or flavones [86]. The brown algal polyphenols were investigated in an SKH-1 hairless mouse skin model with UVB-induced skin carcinogenesis. This in vivo report demonstrated that both dietary feeding and topical treatment of brown algal polyphenols has suppressed cyclooxygenase-2 (COX-2) expression and cell proliferation [87]. These results suggest the role of brown algae polyphenols, phlorotannins, as potential cancer chemo-preventive agents against photo-carcinogenesis and other adverse effects of UVB exposure. That reveals these compounds may be used as active ingredients in drugs or cosmetic/cosmeceutical formulations, like in sunscreen or anti-aging creams [87].

Marine brown algae-derived phlorotannins have also been investigated for their human beneficial aspects that include hypoallergenic, anti-inflammatory, and hyaluronidase inhibitory activities. In vitro studies with the methanol extracts from marine brown algae *Eisenia arborea* have shown inhibition of histamine release from rat basophile leukaemia cells (RBL-2H3) sensitized with anti-dinitrophenyl (DNP) IgE and stimulated with DNP-BSA [88]. Shibata et al. [89] also studied some length-varied phlorotannins obtained from *Ecklonia bicyclis* (as *Eisenia bicyclis*) and *Ecklonia kurome* in their ability to inhibit hyaluronidase activity in vitro. In fact, they proved that those molecules have a stronger inhibitory effect on hyaluronidase than the well-known inhibitors catechins and sodium cromoglycate [89]. 

## 3. Conclusions

This review intended to demonstrate the versatility and the multiple applications of marine macroalgae.

Many products we consume or use daily contain seaweed extracts in their composition, such as ham, ice cream, bottled chocolate drinks, and toothpaste or deodorizers, although most people probably do not even imagine such thing. Nowadays, there is a growing interest in seaweeds due to the recognition of numerous new bioactive compounds. Antioxidants, antimicrobials, anti-inflammatory, anti-aging, anticancer, are just some of its amazing properties to use as pharmaceuticals, cosmeceuticals, nutraceuticals, or even in agriculture or feeding.

There is more and more awareness of sustainable use of natural resources, rather than synthetic and processed products with eventual harmful side effects to the consumer. All the growing interest in these potentialities led to the fostering of macroalgae production, as well as to do research on them. Seaweeds are a resource to maintain and preserve with unique properties.

## Figures and Tables

**Table 1:** Examples of seaweed-derived food products.

Seaweed species	Extract	Product(s)	Reference(s)
Chlorophyta (green seaweed)			
*Cladophora* sp.	Fresh or dry	Sea vegetable	[14]
*Ulva* (formerly *Enteromorpha*) sp., *Monostroma* sp.	Fresh or dry	Sea vegetable	[14]
*Ulva pertusa*	Fresh or dry	Sea vegetable (known as green nori)	[15]
*Ulva rigida*	Fresh or dry	Sea vegetable Seasoning in ready to eat canned fish Added to marine salt	[16]
Ochrophyta, Phaeophyceae (brown seaweed)			
*Fucus vesiculosus*	Extract ^1^ Fresh or dry	Incorporated in honey Seasoning in ready to eat canned fish Added to marine salt	[16,17]
*Himanthalia elongata*	Dry	Sea vegetable Pasta	[18,19]
*Himanthalia elongata*, *Undaria pinnatifida*	Fresh or dry (whole or in flakes) Sodium alginate extract	Wrap Tartar with olives Kelp noodles	[14,18,19,20]
*Sargassum fusiforme*	Fresh or dry	Sea vegetable	[15]
*Undaria pinnatifida*	Fresh or dry	Sea vegetable Pasta	[14,19]
Rhodophyta (Red seaweed)			
*Chondrus crispus*	Fresh or dry	Sea vegetable	[14,16]
*Meristotheca papulosa*	Fresh or dry	Sea vegetable	[14]
*Palmaria palmata*	Fresh or dry (whole or in powder)	Sea vegetable Bacon substitute	[16,18]
*Pyropia* spp. (*P. dioica, P. tenera, P. yezoensis*), *Porphyra umbilicalis*	Fresh or dry	Sea vegetable Nori sheets Laverbread Crispy nougat Crispy thins/snacks Added to marine salt	[15,16,19,21]

^1^ The extract/compound used in the product(s) is not specified in the reference.

**Table 2:** Phycocolloids utilized in food industries and its properties.

Seaweed species	Compound	Product(s)	Properties	Reference(s)
Rhodophyta (red seaweed)				
*Gigartina skottsbergii*	Carrageenan: [A] lota [B] Kappa [C] Lambda	yoghurts, flans, jellies, ice creams, meat products (ham)	[A] and [B]—gelling [C]—thickening/viscosifier	[9,25,26]
*Gelidium* sp., *Gracilaria* sp., *Pterocladiella* sp.	Agar	vegetal jelly	Gelling	[7,9]
		Ochrophyta, Phaeophyceae (brown seaweed)		
*Lessonia* spp., *Macrocystis* sp.	Alginate	yoghurts, ice creams	Emulsifying, Gelling, Stabilizer	[27]

**Table 3:** Cosmetical products containing seaweed parts or extracts.

Seaweed species.	Extract	Product(s)	Properties	Reference(s)
Chlorophyta (green seaweed)				
*Caulerpa lentillifera*	Extract (Rich in unsaturated fatty acids and vitamin A and C)	Hair and skin care products (shampoo, shower gel, soaps, lotions)	Moisturizing; anti-aging; whitening/lightening agent	[58]
*Cladophora glomerata*	Extract (Rich in unsaturated fatty acids and polyphenols)	Skin care products (emulsion, cream, lotion)	Moisturizing; anti-aging	[48,50]
*Codium tomentosum*	Extract Codiavelane^®^	Skin care products (creams, lotions)	Anti-aging; moisturizing	[49,52,59]
*Monostroma* sp.	Extract (rich in water-soluble polysaccharides) Extract ^1^	Skin care products (e.g. slimming and anti-cellulitis formulations) Hair and nails care products (hair and nails growth) Facial Mask	Moisturizing; anti-inflammatory agent; anti-aging	[49]
*Ulva compressa* (as *Enteromorpha compressa*)	Extract ^1^	Skin care products (creams, lotions)	Moisturizing	[59,60]
*Ulva lactuca*	Hydrolysed extract Aosaine^®^ (three-quarters of aosaine consists of amino acids very similar those responsible for the skin’s elasticity)	Skin care products (creams, lotions)	Anti-aging (anti-wrinkle and collagen stimulation)	[49,56,58]
*Ulva* spp.	Aqueous extract (rich in ulvans) Extracts ^1^	Skin care product (creams, lotions) Bath salts (thalassotherapy kit)	Moisturizing; whitening/lightening; antioxidative; chelating; anti-inflammatory; calming	[61,62,63]
Ochrophyta, Phaeophyceae (brown seaweed)				
*Alaria esculenta*	Extract (rich in fatty acids and trace elements)	Skin care products (creams, lotions)	Moisturizing; anti-aging	[59,64]
*Ascophyllum nodosum*	Extract ^1^	Skin care product (cream)	Anti-ageing; skin softness and elasticity restoring	[59]
*Bifurcaria bifurcata*	Extract ^1^	Bath salts, gel and facial mask (thalassotherapy kit)	Exfoliant; detoxifying; nourishing	[61]
*Fucus serratus*	Extract ^1^	Oral-care product	Protecting agent (reduces gingivorrhagia)	[58]
*Fucus spiralis*	Extract ^1^	Facial mask and (imperfection corrector) gel	Skin purification; oiliness and pore dilatation reduction	[65]
*Fucus vesiculosus*	Extract (rich in muco-polysaccharides)	Slimming and anti-cellulitis cosmetic formulations Facial Mask	Skin softness and elasticity properties; exfoliant; brightening; detoxifying	[49,58]
*Halopteris scoparia*	Extract (rich in anti-oxidative polyphenols, cytokines and betaines)	Skin care products (cream, lotion)	Skin softness and elasticity restoring	[59]
*Sargassum fusiforme* (as *Hizikia fusiforme*)	Extract ^1^	Skin care products (creams)	Whitening/lightening;	[58]
*Laminaria digitata*	Extract (rich in trace elements, like iodine)	Skin care products (lotions, anti-cellulitis formulations)	Anti-aging (prevent lines and wrinkles. Collagen and elastin stimulation); anti-cellulitis; moisturizing	[58,59,60]
*Laminaria hyperborea*	Extracts ^1^	Skin care product (cream) Facial masks	Anti-aging; moisturizing; anti-acne	[59,65]
*Laminaria ochroleuca*	Extract ANTILEUKINE 6™ Extracts ^1^	Hair and skin care products (body lotion, shampoo and conditioner)	Anti-aging; sun-protector; anti-acne; moisturizing	[56,59,65]
*Saccharina latissima* (as *Laminaria saccharina*)	Extract (w/ hyaluronic acid and polysaccharides; sodium and potassium ions; phlorotannins (polyphenols))	Skin care product (cream)	Antioxidant; anti-aging; anti-blemishes	[59]
*Macrocystis pyrifera*	Extract (rich in polysaccharides) Extract ^1^	Skin care product (anti-aging balm)	Moisturizing; antioxidant; anti-aging; anti-blemishes	[44,59]
*Pelvetia canaliculata*	Extracts ^1^	Hair and skin care products (creams, lotions, shampoo)	Moisturizing; anti-aging (anti-wrinkle and collagen stimulation)	[58,59]
*Saccharina japonica*	Polysaccharide extract (rich in fucoidan)	Skin care products (anti-cellulitis formulations)	Moisturizing; anti-aging; anticellulite	[52,53,66]
*Kjellmaniella crassifolia*	Fucoidan extract	Hair and skin care products (creams, lotions, shampoo)	Moisturizing; anti-aging; nourishing; preventing hair loss	[67]
*Sargassum muticum*	Extract (rich in proteins)	Skin care products (creams, lotions)	Anti-aging (anti-wrinkle, antioxidant, and collagen stimulation. Reduce skin damage caused by UVB and chemical stress)	[59]
*Undaria pinnatifida*	Extract ^1^ Powder, whole leaf and extract forms (rich in fucoidan)	Skin care products (aromatherapy oil; face and body oil; body scrub)	Anti-aging (anti-wrinkle); whitening/lightening; moisturizing; nourishing	[44,58,59]
Rhodophyta (red seaweed)				
*Asparagopsis armata*	Extract ASPAR’AGE™	Skin care products (creams)	Moisturizing; anti-aging	[56]
*Chondrus crispus*	Extracts ^1^ Powder	Hair and skin care products (lotions; creams; make-up removers; body scrub; shampoo; conditioner) Lipsticks and deodorants Algae and sea salt soap	Moisturizing; cleaning; exfoliant; Emulsifier and thickener; cleaning; exfoliant	[58,59,65,68]
*Corallina officinalis*	Extract ^1^	Skin care product (cream)	Anti-redness	[59]
*Gelidium corneum* (as *Gelidium sesquipedale*)	Extract (rich in minerals, trace elements and amino acids)	Skin care product (lotion)	Skin softness and elasticity restoring	[59]
*Gigartina skottsbergii*	Powder, whole leaf and extract ^1^ Extract (rich in polysaccharides, vitamins and minerals)	Bath and skin care products (mineral-rich seaweed bath soak)	Moisturizing; whitening/lightening	[44]
*Gracilaria conferta*	Extract ^1^	Skin care products (creams)	Moisturizing; nourishing	[58]
*Palmaria palmata*	Extract ^1^	Skin care products (Facial clarifier gels and emulsions)	Skin clarification (reduction of pigmentation imperfections), and uniformization (skin grain homogenization)	[65]
*Pyropia tenera* (as *Porphyra tenera*)	Extract ^1^	Skin care products (creams)	Sun protector	[58]
*Porphyra umbilicalis*	Extract ^1^	Skin care products (cream; facial scrub masks)	Moisturizing; exfoliant; brightening; detoxifying	[59,69]
*Vertebrata lanosa* (as *Polysiphonia lanosa*)	Extract ^1^	Skin care products (creams)	Moisturizing; nourishing	[52,65]

^1^ The extract/compound used in the product(s) is not specified in the reference.
